# Supplementary material for: MARCH6 and TRC8 facilitate the quality control of cytosolic and tail‐anchored proteins
Source: EMBO Rep. 2018 Mar 8;19(5):e45603. doi: 10.15252/embr.201745603 (PMC5934766; doi:10.15252/embr.201745603)
Supplement: Supplementary file 1 — Appendix [file EMBR-19-e45603-s001.pdf]

Appendix

Table of Contents

Appendix Figures

Figure S1: Gp78 is not required for the degradation of mCherry-CL1 in Hela Cells.

Figure S2: Indel formation in the MARCH6 sgRNA targeted HeLa clones.

Figure S3: Reconstitution of the UBE2G2, TRC8 or MARCH6 KO HeLa cells restores mCherry-CL1 degradation.

Figure S4: Lysine-48 polyubiquitin linkages promote the degradation of mCherry-CL1.

Figure S5: Depletion of Bag6 does not alter mCherry-CL1 levels.

Figure S6: MARCH6 and TRC8 are required for the degradation of selected ER proteins following SPP mediated intramembrane proteolysis.

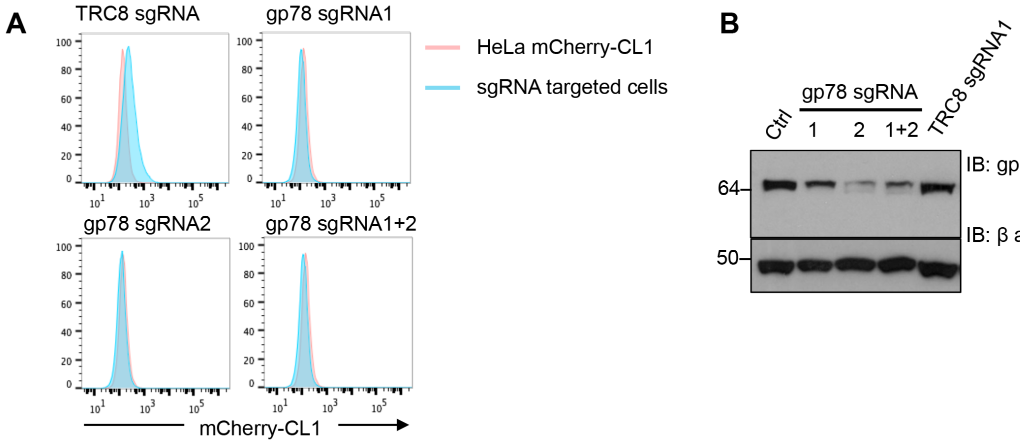

Figure S1: Gp78 is not required for the degradation of mCherry-CL1 in Hela Cells.

(A, B) HeLa mCherry-CL1 cells (red) were transduced with Cas9 and sgRNA targeting TRC8 or gp78 (blue) and mCherry fluorescence measured by flow cytometry after 7 days **(A)**. Depletion of Gp78 was confirmed by immunoblot **(B)**. Two different sgRNA (sgRNA 1 or 2) targeting gp78 were used either by themselves or in combination. β-actin levels served as a loading control.

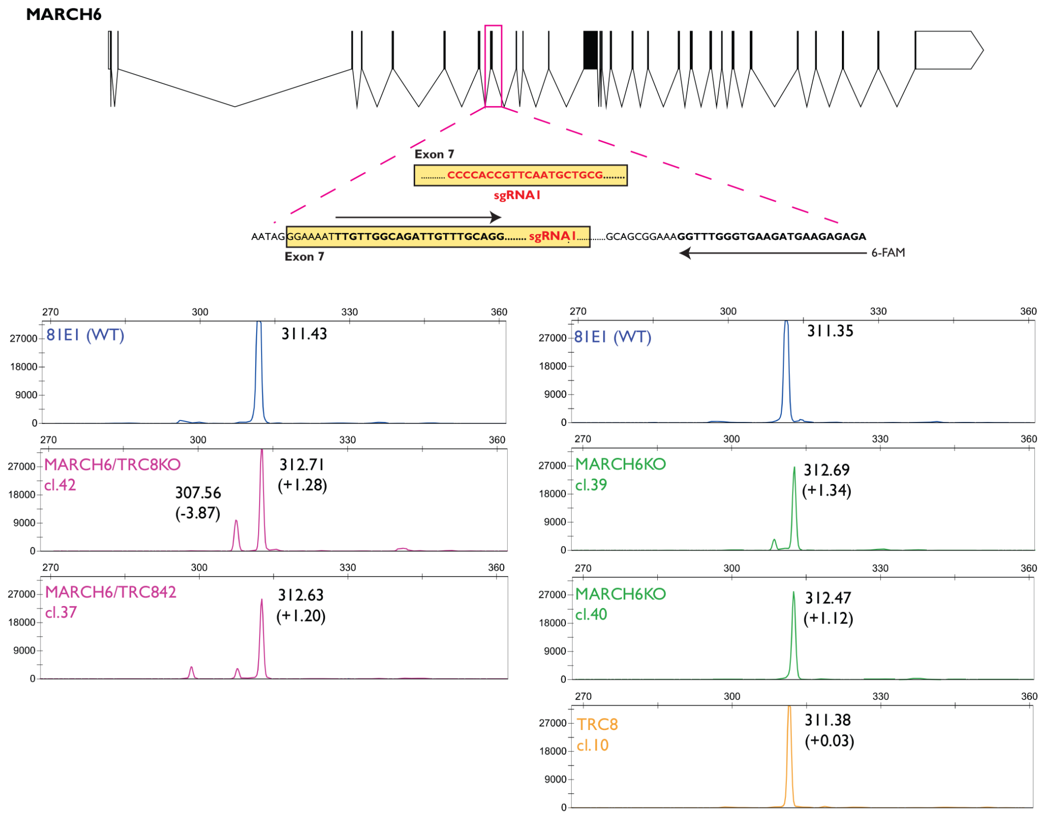

Figure S2: Indel formation in the MARCH6 sgRNA targeted HeLa clones.

Indel formation in the MARCH6 sgRNA targeted clones was confirmed by fluoresecence PCR to identify small frame-shifts. Genomic DNA was extracted from the clones and amplified with MARCH6 specific primers. Indel formation was analysed by capillary electrophoresis using the 3730xL Genetic Analyser (Applied Biosystems) and visualised in Gene Mapper 5 (Applied Biosystems).

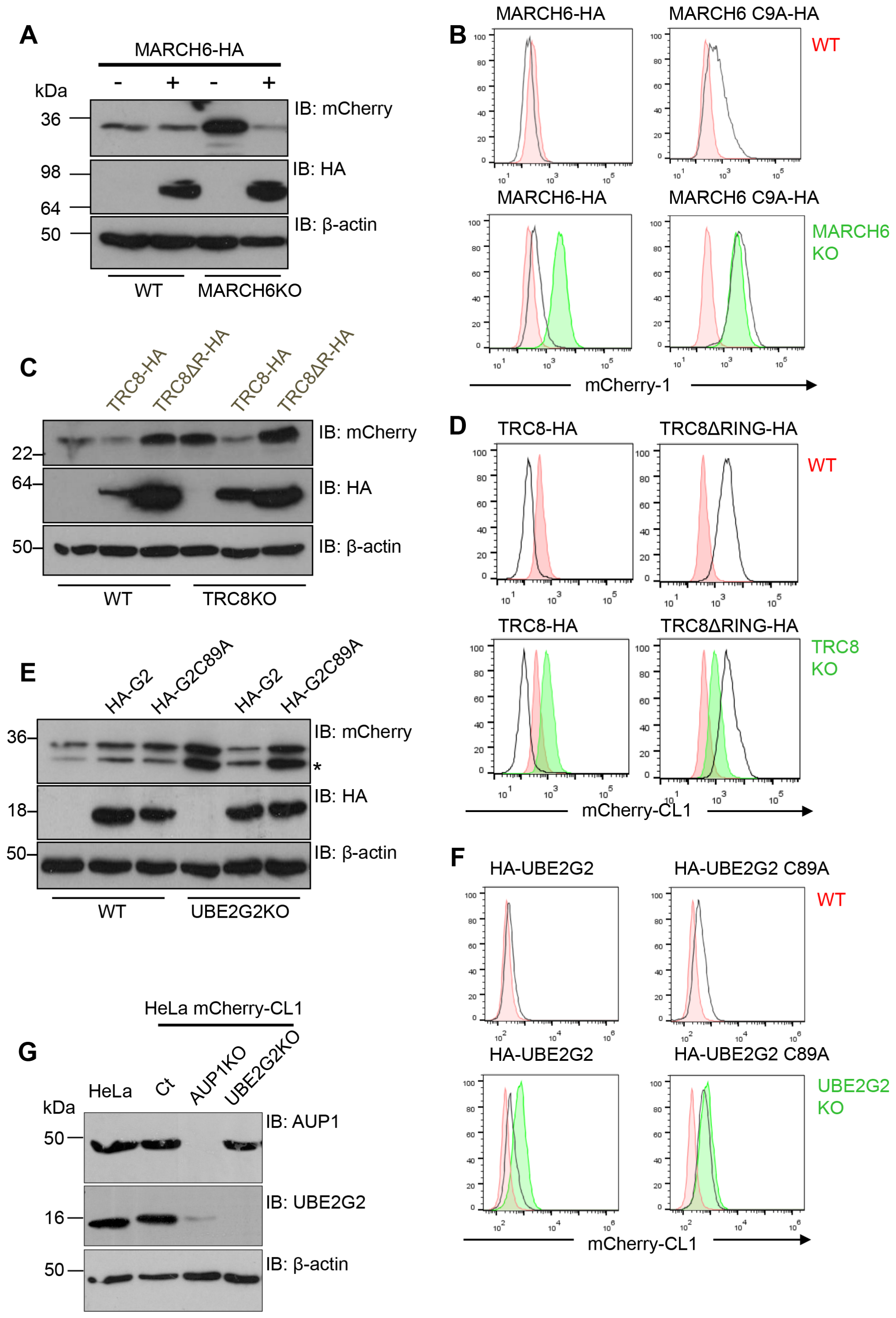

Figure S3: Reconstitution of the UBE2G2, TRC8 or MARCH6 KO HeLa cells restores mCherry-CL1 degradation.

(A-CF) Reconstitution of the MARCH6 **(A, B)**, TRC8, **(C, D)** or UBE2G2 **(E, F)** null HeLa mCherry-CL1 reporter cells by overexpression with the transiently transfected respective HA tagged proteins. MCherry-CL1 levels were measured by immunoblot **(A, C, E)** 48 hr after transfection or by flow cytometry **(B, D, F)**. For flow cytomtery, mCherry-CL1 levels in the parent reporter cells (red), null

clones (green) and reconstituted cells (black line) are shown. Catalytic inactive mutants (MARCH6 C9A-HA, HA-UBE2G2 C89A, and TRC8ΔRING-HA) were also overexpressed in the respective null clones. **(G)** UBE2G2 protein levels in the AUP1 KO or UBE2G2 KO HeLa mCherry-CL1 clones were measured by immunoblot. *G2=UBE2G2, Btz=Bortezomib.*

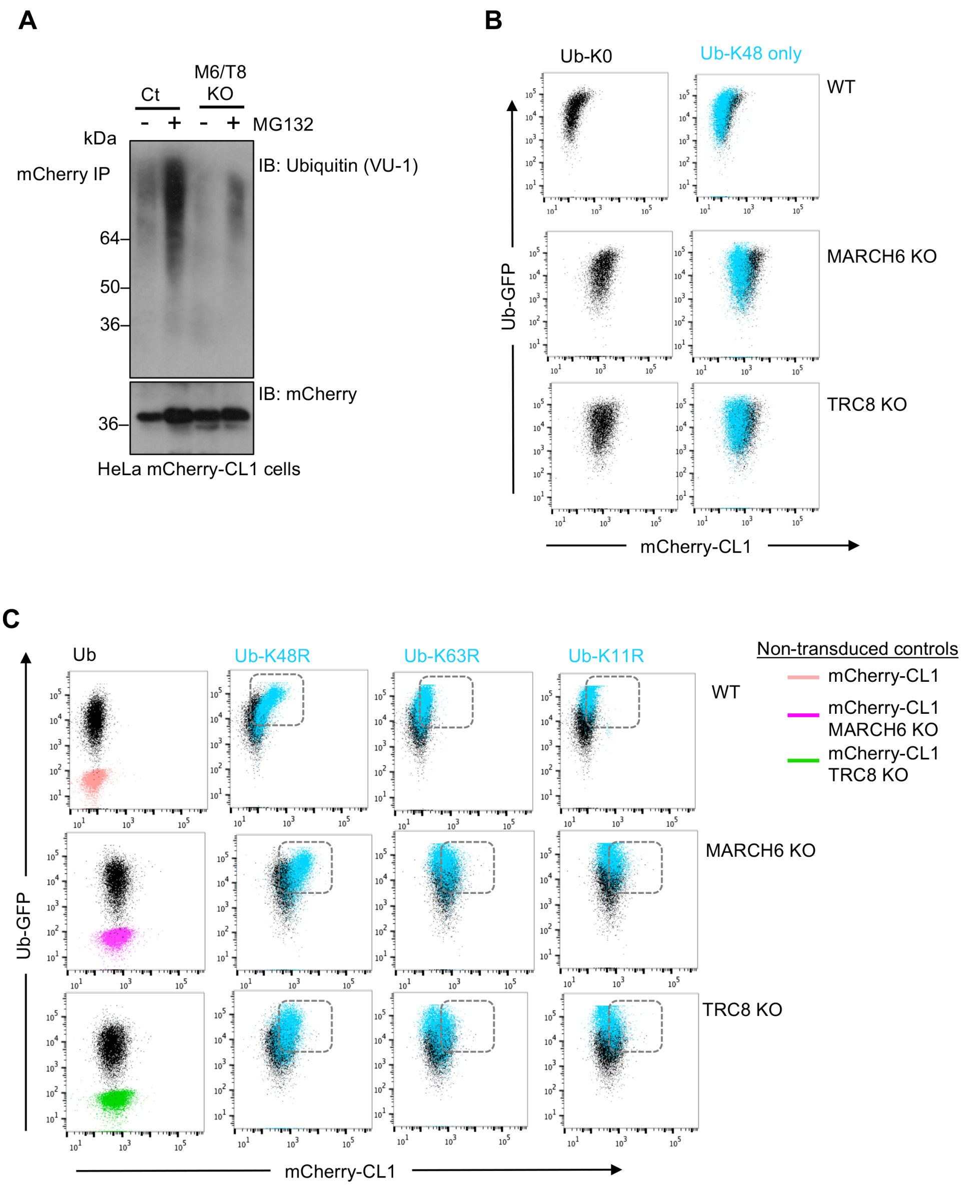

Figure S4: Lysine-48 polyubiquitin linkages promote the degradation of mCherry-CL1.

(A) Ubiquitination of mCherry-CL1. Wildtype or combined MARCH6/TRC8 null cells were treated with the proteasome inhibitor MG132 (50μM) for 2 hr prior to lysis in 1% Digitonin. An mCherry antibody was used to immunoprecipitate mCherry-CL1, and mCherry-CL1 or ubiquitin levels (VU-1 antibody) measured by immunoblot. **(B, C)** Competitive inhibition of mCherry-CL1 ubiquitination using exogenous ubiquitin mutants. Wildtype, MARCH6 null or TRC8 null Hela mCherry-CL1 cells were transduced with ubiquitin-GFP expressing no lysines (Ub-KO, black) in comparison to a ubiquitin-GFP with a single lysine 48 (Ub-K48 only), and mCherry levels measured by flow cytometry **(B)**. Wildtype, MARCH6

null or TRC8 null Hela mCherry-CL1 cells transduced with ubiquitin-GFP (black) or single lysine ubiquitin mutants (Ub-K48R, Ub-K63R or Ub-K11R) (blue) **(C)**. Grey boxes indicate the stabilisation of mCherry-CL1 in the cells with high levels of Ub-K48R. GFP and mCherry levels in the respective control cells are also shown.

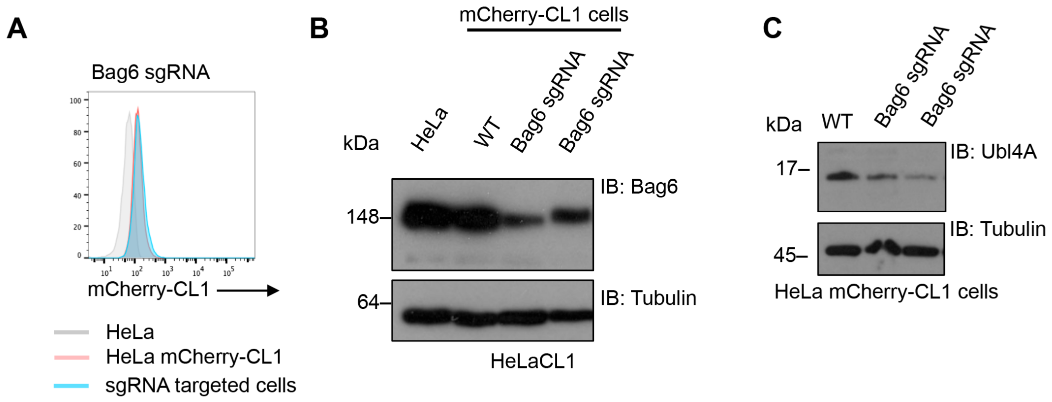

Figure S5: Depletion of Bag6 does not alter mCherry-CL1 levels.

(A-C) CRISPR/Cas9 depletion of Bag6 in HeLa mCherry-CL1 cells. Cells were transduced with pooled sgRNA targeting Bag6 (three guides), and mCherry levels measured by flow cytometry after 7 days (shaded blue) **(A)**. Basal mCherry-CL1 levels are shown (red). Bag6 depletion was confirmed by immunoblot **(B)** and by decreased levels of Ubl4A **(C)**. Duplicate experimental replicates are shown **(B, C)**.

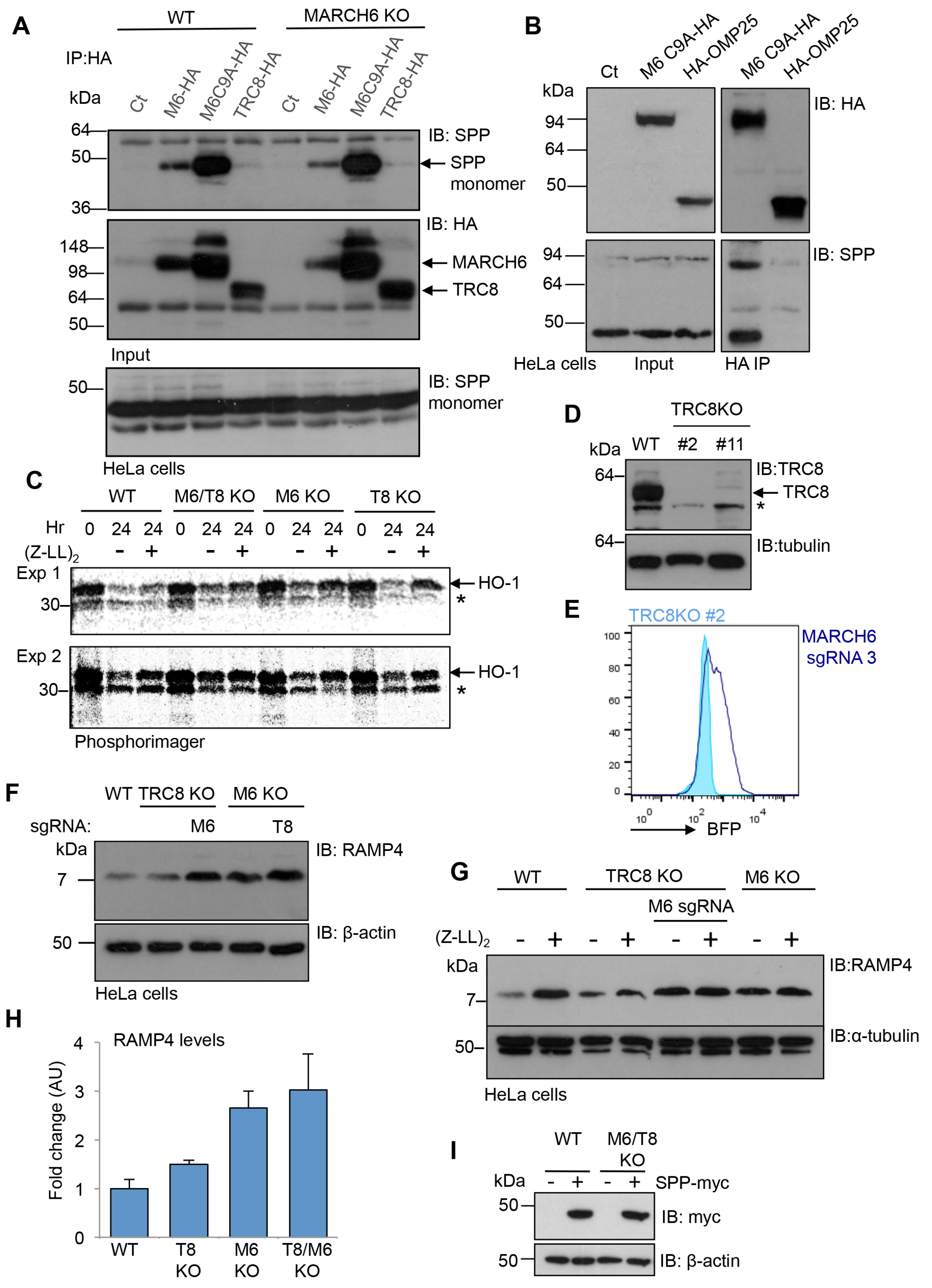

Figure S6: MARCH6 and TRC8 are required for the degradation of selected ER proteins following SPP mediated intramembrane proteolysis.

(A, B) Immunoprecipitation of MARCH6-HA, TRC8-HA, or MARCH6 C9A-HA with endogenous SPP in HeLa cells. **(A)** Hela mCherry-CL1 cells (WT) or the MARCH6 KO clone were lentivirally transduced with MARCH6-HA, MARCH C9A-HA or TRC8-HA, lysed in 1% Digitonin, and immunoprecipitated for HA. Resin bound

proteins were washed with 0.5% Digitonin and immunoblotted for SPP. SPP levels in the input samples are also shown. **(B)** Hela cells (Ct) or those stably expressing MARCH6 C9A-HA were lysed in 1% Digitonin and immunoprecipitated with an HA antibody as described. Cells expressing HA-OMP25 (a mitochondrial protein) were used as an HA tagged control. Protein levels in the detergent lysate inputs are also shown (left). **(C)** [^{35}S]methionine/cysteine-radiolabelling of HO-1 in Hela mCherry-CL1 cells, MARCH6 KO, TRC8 KO or the combined MARCH6/TRC8 null cells. Cells were pulse radiolabelled for 10 min, and immunoprecipitated for HO-1 from detergent lysates at the times indicated. HO-1 levels were measured by phosphorimager. Two experiments are shown. *Represents non-specific band observed in HeLa mCherry-CL1 cells. **(D)** Representative immunoblot for confirmation of TCR8 protein loss in TRC8 null clones. TRC8 null clones were isolated from HeLa cells transduced with Cas9 and TRC8 sgRNA. *Non-specific band. **(E)** Representative transfection of Cas9 and MARCH6 a TRC8 null clone, using BFP levels as a measure the transfection efficiency for MARCH6 sgRNA. **(F)** Immunoblot of endogenous RAMP4 in HeLa cells, or clonal populations of HeLa TRC8 or MARCH6 deficient cells. The null clones were also transiently transfected with Cas9 and sgRNA to TRC8 or MARCH6 to generate combined TRC8/MARCH6 deficient HeLa cells. β-actin served as a loading control. **(G)** Immunoblot of RAMP4 in wildtype, MARCH6 null, TRC8 null or MARCH/TRC8 null cells with or without (Z-LL)$_2$ ketone treatment. Tris-Tricine gels were used to resolve RAMP4 protein levels. α-tubulin served as a loading control. **(H)** Quantification of RAMP4 levels in the MARCH6 null, TRC8 null or MARCH6/TRC8 deficient cells (Mean ± SEM, n=3). **(I)** Immunoblot of overexpressed SPP-myc levels in wildtype HeLa cells or combined MARCH6/TRC8 null cells. β-actin served as a loading control. *WT=wildtype HeLa cells, Ct=control, M6 =MARCH6, T8=TRC8.*
